# Patients’ perception of coercion with respect to antipsychotic treatment of psychotic disorders and its predictors

**DOI:** 10.1007/s00127-021-02083-z

**Published:** 2021-04-27

**Authors:** Sophie Hirsch, Nancy Thilo, Tilman Steinert, Erich Flammer

**Affiliations:** Centres for Psychiatry Suedwuerttemberg, Clinic for Psychiatry and Psychotherapy I, Ulm University, Weingartshofer Str. 2, 88214 Ravensburg, Germany

**Keywords:** Perceived coercion, Patient and staff perspectives, Insight into illness, Attitude towards medication, Symptom severity

## Abstract

**Purpose:**

The present study investigates perceived coercion in psychiatric inpatients under prescribed antipsychotic medication without a court order. The objective of this study was to investigate whether and to what extent involuntary and voluntary inpatients feel coerced to take their medication and which factors affect perceived coercion.

**Methods:**

Voluntarily and involuntarily admitted patients (55 and 36, respectively) were interviewed about the extent of perceived coercion. In addition, socio-demographic and clinical data were collected. The Admission Experience Scale (aAES) was used to assess perceived coercion concerning medication. To measure insight into illness, attitude towards medication, and symptom severity, we used a questionnaire on insight into illness (FKE-10), the Drug Attitude Inventory (DAI-10), and the Brief Psychiatric Rating Scale (BPRS-24), respectively.

**Results:**

Voluntarily treated patients experienced significantly less coercion when taking prescribed medication in inpatient settings than involuntarily treated patients. The experience of coercion was not related to socio-demographic or clinical variables nor to the BPRS-24 score, but to insight into illness and attitude towards medication. Patients who had experienced at least one coercive measure during the index hospital stay showed a higher level of perceived coercion.

**Conclusion:**

Perceived coercion related to medication is dependent on insight into illness and experience of previous coercive interventions rather than on the severity of psychopathological symptoms. These findings are very similar to a previous study in a forensic psychiatric sample. Having experience of at least one coercive measure seems to be a decisive aspect of the extent of the patients’ perceived coercion.

**Supplementary Information:**

The online version contains supplementary material available at 10.1007/s00127-021-02083-z.

## Introduction

Coercion occurs in about 3–35% of psychiatric admissions worldwide and is one of the oldest and most controversially discussed issues in psychiatry [1, 2]. Coercion occurs in the form of involuntary admission and subsequent involuntary commitment to a hospital, in aspects of informal coercion [3] and, with still higher impact on the patient’s autonomy and personal rights, as seclusion, mechanical or physical restraint, or involuntary medication. Apart from patients with organic disorders, patients with schizophrenic disorders are most frequently affected by coercive measures [4, 5]. This applies particularly for medication, because for these disorders, medication is expected to exert not only tranquilizing but also therapeutic effects [6, 7]. Involuntary medication covers a considerable range from informal coercion (e.g., weekend leave only when patients take medication), to open psychological pressure (threatening with an injection or seclusion if oral medication is refused) and through to intramuscular administration of medication by force. Substances used are virtually exclusively benzodiazepines for sedation or rapid tranquilization and antipsychotics for rapid tranquilization and antipsychotic treatment, without any clear discrimination. For long-term antipsychotic treatment, depot antipsychotics also can be administered on an involuntary basis.

Coercive measures of all kinds, whether experienced by the patient or observed by others, are very stressful for the patient. This distress is closely linked to a negative assessment of psychiatric treatment in general and the experience of humiliation and feelings of shame and self-loathing [8–12].

Coercive treatment is caught in the dilemma between respecting the patient’s autonomy and the doctor’s duty to act in the patient’s best interest [13, 14]. After two decisions by the German Constitutional Court in 2011 [15, 16] that had judged previous regulations in public law and civil law to be unconstitutional, new regulations for involuntary treatment within the scope of involuntary commitment and guardianship have been implemented, setting up a high legal threshold to obtain permission for the use of medication against a patient’s expressed will. Administering medication by coercion since then is only possible in cases of acute emergency or after a comprehensive review by an independent psychiatrist, followed by a judge’s decision. As a consequence, formal involuntary medication is indeed rare in German psychiatric and forensic hospitals [17], but the prevalence rates of informal coercion are still estimated to be quite high [18–20].

In recent years, the subjective experience and evaluation of coercion from the perspective of patients and staff has been the subject of several studies (e.g., [10, 12, 21]). The subjective experience of coercion can make it difficult to build a trusting therapeutic alliance [18, 22–25].

A study in two forensic hospitals in southwest Germany on the perceived coercion regarding prescribed antipsychotic medication showed that although only a few patients were affected by emergency rapid tranquilization or compulsory medical treatment according to a court order, many patients felt compelled to take their antipsychotic medication [26]. The extent of feeling coerced to take the prescribed medication was affected mainly by the patient’s attitude towards medication and the degree of insight into illness but less so, and not significantly, by symptom severity. Against this background, we wanted to extend these previous findings to general psychiatry.

The aim of the study was to investigate to what extent patients in general psychiatry, who are not forced to take their medication by law, perceive coercion and if there are any differences between patients admitted against their will and patients voluntarily admitted.

The following questions will be in focus:

1. To what extent do patients who are hospitalized voluntarily or involuntarily perceive coercion in the intake of prescribed antipsychotics?
2. Does the extent of perceived coercion when taking medication depend on socio-demographic characteristics, clinical characteristics, and psychopathological symptoms?
3. Is there an association between perceived coercion and insight into illness and attitude to medication?
4. What characteristics can be attributed to a high degree of perceived coercion when taking antipsychotics in inpatient settings?

## Methods

### Inclusion criteria

Inclusion criteria were age 18–65 years, a main psychiatric diagnosis of schizophrenia, schizotypal or delusional disorders (ICD-10 diagnosis of category F20–29), inpatient treatment, and written informed consent to participate in the study.

### Exclusion criteria

Exclusion criteria were intellectual disability (ICD-10 diagnosis of category F70–79), insufficient knowledge of the German language, and no prescription of antipsychotic medication.

### Participants

Between September 2017 and June 2019, 91 general psychiatric patients from two general psychiatric hospitals in Weissenau and Friedrichshafen were interviewed, of which 36 were involuntarily admitted.

### Study design

We used an analytical observational cross-sectional design.

### Recruitment of participants

Participants were recruited on four wards of two general psychiatric hospitals. They were identified according to the inclusion criteria, with the help of the staff of participating wards. After written consent to participate in the study, the patients were interviewed face to face by NT.

### Instruments

#### Socio-demographic data and data on coercive measures, diagnoses, and legal status

Socio-demographic data and data on coercive measures, diagnoses, and legal status (i.e., voluntary vs. involuntary hospital stay) were taken from the electronic medical records.

#### Adapted Admission Experience Scale (aAES*)*

To assess the extent of perceived coercion with respect to prescribed antipsychotic medication, we used an adapted version of the MacArthur Admission Experience Survey [26, 27].

#### Questionnaire on insight into illness (Fragebogen zur Krankheitseinsicht, FKE-10)

A questionnaire about insight into illness (FKE-10) was used to measure the self-assessment of patients with regard to their own symptoms and their need for treatment with medication [28].

#### Drug Attitude Inventory (DAI-10)

The attitude of psychotic patients towards their illness and antipsychotic medication was examined using the Drug Attitude Inventory [29].

#### Brief Psychiatric Rating Scale, Extended Version (BPRS-24)

The BPRS was used to measure changes in psychopathology in pharmaceutical studies covering affective symptoms, positive and negative symptoms of schizophrenia, activation, and disorganization [30].

### Data collection

Socio-demographic data and data on coercive measures, diagnoses, and legal status (i.e., voluntary vs. involuntary hospital stay) were provided by the hospitals’ medical control department.

The aAES, FKE-10, DAI-10, and BPRS-24 were presented to the participants for answering in the presence of the interviewer. For any comprehension difficulties, the participants could ask the interviewer. The interviewer assessed the participants’ symptoms based on the BPRS-24.

### Calculation

Data analysis was carried out using the statistics and analysis software IBM SPSS Statistics Version 24.0. Missing values up to a maximum of 30% per scale were replaced by personal mean imputation [31]. Depending on the data, both parametric (*t* test, analysis of variance) and non-parametric tests (Mann–Whitney *U* test, Kruskal–Wallis test) were used to assess differences between groups. Effect sizes were calculated as eta for frequencies and as Cohen’s *d* for metric variables. Cohen’s *d* can be rated as small (0.2), medium (0.5), and large (0.8) [32]. For their calculations, the actual number of measurement values was taken as a basis. To assess the correlations between variables, Pearson’s correlation coefficients were calculated.

To assess possible predictors of perceived coercion measured by the aAES, linear regression models were fitted. Possible predictors that showed significant associations with the aAES in bivariate analyses were entered into the models. To assess the degree, to which predictors explain the variation of perceived coercion, we calculated the adjusted *R*^2^. The adjusted *R*^2^ can be interpreted as percent of variance explained. Linearity was assessed by visual inspection of the plots of observed versus predicted values. Homoscedasticity was assessed by visual inspection of the probability–probability plot of observed versus predicted cumulated probability of the residuals. The normal distribution of residuals was tested with a Kolmogorov–Smirnov test. Multicollinearity was tested with bivariate correlations between possible predictors. Analyses were carried out with and without imputations. Results without imputation are presented as Supplementary Material. Post hoc power analysis for multiple regression was carried out using the Post hoc Statistical Power Calculator for Multiple Regression [33]. We also performed analyses of complete versus incomplete cases. Complete cases were defined as cases with complete data on the aAES, FKE-10, DAI-10, voluntariness of hospital stay, and coercive measures.

## Results

Out of 91 participants, 35 (38.5%) were female. The mean age was 38.9 years (SD = 13.0). Table 1 shows the socio-demographic characteristics of the participants.

**Table 1:** Socio-demographic characteristics of the participants

Gender	
Female	35 (38.5%)
Male	56 (61.5%)
Marital status	
Single	66 (72.5%)
Married/partnership	14 (15.5%)
Widowed/divorced	10 (11.0%)
Unknown	1 (1.1%)
Migration background	
Yes	25 (27.5%)
No	64 (70.3%)
Unknown	2 (2.2%)
German as first language	
Yes	77 (84.6%)
No	11 (12.1%)
Unknown	3 (3.3%)
Living condition	
Non-assisted living	61 (67.0%)
Assisted living	21 (23.1%)
Homeless	5 (5.5%)
Unknown	4 (4.4%)
Vocational qualification completed	
Yes	51 (56.0%)
No	32 (35.2%)
Unknown	8 (8.8%)
Number of previous psychiatric hospital stays	
None	1 (1.1%)
One	9 (9.9%)
2–5	37 (40.7%)
> 5	32 (35.2%)
Unknown	12 (13.2%)
Voluntary hospital stay	
Yes	55 (60.4%)
No	36 (39.6%)
Psychiatric main diagnosis according to ICD-10	
F20	71 (78.0%)
F25	12 (13.2%)
F31	8 (8.8%)
Experience of at least one coercive measure in lifetime	59 (64.8%)
Experience of at least one coercive measure during index hospital stay	37 (40.7%)

Voluntarily treated participants did not differ significantly from involuntarily treated participants in terms of socio-demographic characteristics, diagnoses, and number of previous hospital stays. Significant differences were found between involuntarily and voluntarily treated participants in terms of experience of coercive measures, insight into illness (FKE-10, large effect), attitudes towards medication, need for treatment (DAI-10, medium effect), and psychopathological symptoms (BPRS-24, modest effect) (Table 2). Perceived coercion when taking prescribed antipsychotic drugs was significantly higher in involuntarily treated participants with a large effect size (Table 2).

**Table 2:** Differences between involuntarily and voluntarily treated participants

	Participants		Effect size eta/Cohen’s *d*^(1)^
	Involuntarily treated (*n* = 36) Number (%)/mean (SD)	Voluntarily treated (*n* = 55) Number (%)/mean (SD)	
Experience of at least one coercive measure in lifetime	32 (88.9%)	27*** (49.1%)	0.41
Experience of at least one coercive measure during index hospital stay	25 (69.4%)	12*** (21.8%)	0.47
aAES	11.5 (5.2)	6.5*** (4.9)	− 1.0
FKE-10	23.8 (9.7)	36.7*** (8.8)	1.4
DAI-10	–2.6 (4.9)	0.71** (5.4)	0.6
BPRS-24	62.1 (18.0)	50.9** (14.9)	− 0.7

(1) Eta for frequencies; Cohen’s *d* for metric variables

^**^p < .01 and ***p < .001; Mann–Whitney *U* test

Age, gender, main psychiatric diagnosis, and number of previous psychiatric hospital stays were not statistically significantly associated with perceived coercion (aAES). Furthermore, marital status, migration background, language, living condition, and vocational qualification showed no statistically significant association with perceived coercion (aAES). Participants who had experienced at least one coercive measure in their lifetime did not express a statistically significant higher perceived coercion (aAES) (mean = 8.9, SD = 5.9) than participants who had not experienced coercive measures (mean = 7.5, SD = 4.7).

Insight into illness (FKE-10), attitude towards medication (DAI-10), and psychopathological symptoms (BPRS-24) showed an association with the extent of perceived coercion (aAES) (Table 3). Participants who had experienced at least one coercive measure during the index hospital stay expressed a statistically significant higher perceived coercion (aAES) (mean = 10.8, SD = 5.6) than participants who had never experienced coercive measures (mean = 6.8, SD = 5.0, *p* < 0.05).

**Table 3:** Correlations with perceived coercion

	aAES
FKE-10	− 0.65**
DAI-10	− 0.70**
BPRS-24	0.24*

*p < 0.05 and **p < 0.01

A linear regression model with perceived coercion (aAES) as outcome variable and insight into illness (FKE-10), attitude towards medication (DAI-10), psychopathological symptoms (BPRS-24), involuntary hospital stay (yes/no), and experience of at least one coercive measure during the index hospital stay (yes/no) as predictor variables was fitted. Regression diagnostics showed no substantial violation of the underlying statistical assumptions.

The regression model reached overall statistical significance (*F*(5,67) = 33.4, *p* < 0.001). Insight into illness, attitude towards medication, and experience of coercive measures were significantly associated with perceived coercion (aAES) and explained a very substantial percentage of variation. The results are shown in Table 4.

**Table 4:** Prediction of perceived coercion

Adjusted *R*^2^ = .69	Standardized beta
FKE-10	− 0.38***
DAI-10	− 0.47***
BPRS-24	− 0.03^n.s^
Coercive measure experienced during index hospital stay	0.23**
Involuntary stay	0.04^n.s^

*n.s.* not significant

**p < 0.01, ***p < 0.001

For all calculations, sensitivity analyses without imputed data showed the same results (Supplementary Material). Incomplete cases did not differ statistically significantly from complete cases with respect to age, gender, diagnoses, number of previous hospital stays, and socio-demographic variables. The groups also did not differ statistically significantly in aAES scores.

Cases with a complete BPRS-24 did not differ statistically significantly from cases with an incomplete BPRS-24 in the aAES scores. Post hoc power analysis for multiple regression showed a statistical power of 1.0.

## Discussion

In this study, involuntarily admitted patients showed substantially higher expression of perceived coercion with respect to taking their prescribed medication than voluntarily admitted participants. Predictors of a higher level of perceived coercion were less insight into illness, negative attitude towards antipsychotic medication, and experience of at least one coercive measure during index hospital stay. These predictors explained more than two-thirds of the variation between the participants. No association was found between experienced coercion and socio-demographic variables. While these results may have been expected, to our mind, the most interesting finding of this study is a negative one: psychopathological symptoms as measured by the BPRS-24 showed a weak statistical correlation with the extent of perceived coercion in bivariate correlations; however, the multivariate model yielded no more significance for this variable. This finding challenges the deep-rooted belief of psychiatrists that refusal of medication in psychotic disorders should be considered a symptom of the disorder itself that disappears during successful treatment, thereby legitimizing coercion, especially forced medication.

Furthermore, our results are fully in line with results from a previous study in a forensic psychiatric sample using the same method [26]. It is noteworthy that two independently conducted investigations in different populations with regard to the legal basis of treatment have led to almost identical results. Very similar to the results of the current study in general psychiatry, in the forensic sample, 56% of the variance of perceived coercion could be explained by attitude towards medication (DAI-10), insight into illness (FKE-10) and, to a small but significant extent, by symptom severity measured by the Positive and Negative Syndrome Scale (PANSS). As in this study, socio-demographic variables had no significant impact. This high accordance of findings supports the validity and robustness of the results.

Other studies also provide empirical evidence in the same direction. A study of 30 patients with schizophrenia showed that the attitude towards medication did not change, despite improvement of their psychopathological symptoms [34]. A negative attitude towards medication is not only a psychiatric phenomenon but also a problem in the treatment of chronic physical diseases such as diabetes and high blood pressure [35, 36]. This confirms the assumption that a negative attitude towards medication does not represent a symptom of psychotic disorders.

A systematic review revealed a significant correlation between attitude towards medication, insight into illness, and non-adherence in psychiatric patients, regardless of diagnosis and stage of disease [37]. The consequences for the construct of ‘lack of insight into treatment’ are considerable if lack of insight is regarded as a basic personal attitude and not as a mere epiphenomenon of a disease, it seems less justifiable to override the patient’s expressed will.

Further evidence for the hypothesis that negative attitude towards medication is more a personality-related feature than a disease symptom comes from a recent qualitative study [38]. The authors conducted 32 qualitative interviews with patients subjected to forced medication and also with their relatives, psychiatrists, and nurses. They found that the professionals considered the patients’ refusal to take medication to be an aspect of their disease and considered medication as a necessary treatment, whereas patients and their relatives sorted the refusal into a coherent biographical perspective. For many patients, refusing to take medication was an important aspect of their autonomy and, consequently, they felt deeply violated by subtle or even open coercion. The German Federal Constitutional Court and subsequently the legislator have already taken these considerations into account by increasing the requirements for the approvability of coercive treatment. The results of this study provide additional empirical evidence for this ethical position.

The present study has some methodological limitations. First, it is a cross-sectional study that hardly allows statements about causal relationships. Statements on causal relationships require longitudinal studies, with patients being surveyed at different time points during the course of their illness. It should also be noted that the sample size is rather small, which decreases the statistical power of the analyses. However, this effect might not be too serious, because, on comparing voluntarily and involuntarily treated patients with respect to the perceived coercion, the difference (i.e., effect size) is substantial and therefore less prone to type II statistical error. On the other hand, the non-significant effects of voluntary versus involuntary hospital stay and symptom severity are very small in the regression model, so even a very large sample size would probably be insufficient to protect against a possible type II error.

The present study was conducted in two general psychiatric hospitals in southern Germany, clearly restricting the generalizability of the results. To what extent the results of the study are valid for patient populations in other federal states of Germany or other countries is unclear.

The participants were interviewed regarding the subjectively perceived coercion with respect to the prescribed antipsychotic medication at present and in the past. For this purpose, an already validated instrument, the AES, was used in its adapted form (aAES). However, the aAES still requires independent psychometric validation.

Considering the BPRS-24 values obtained, no seriously ill patients took part in the study. In this study as well as the forensic psychiatry study, participants had to be able to concentrate over a longer period of time, understand questions, form an opinion and formulate answers. These inclusion criteria excluded people with very severe or acute symptoms, as in most other studies, and hence, it is possible that our findings are only valid for people with mild-to-moderate symptom severity.

Due to the fact that participation in the study was voluntary, patients who were particularly dissatisfied with their treatment and perceived a particularly high degree of coercion might not have been included in that convenience sample. On the other hand, for patients who had been admitted involuntarily and wanted to quit treatment as soon as possible, effects such as answers according to social desirability might have been active.

## Conclusions

Perceived coercion related to medication is dependent on insight into illness and experience of previous coercive interventions rather than on the severity of psychopathological symptoms. These findings can be considered to be robust, because they are very similar to a previous study in a forensic psychiatric sample. Having experience of at least one coercive measure seems to be a decisive aspect of the extent of the patients’ perceived coercion.

## Supplementary Information

Below is the link to the electronic supplementary material.

Supplementary file1 (DOCX 17 KB)

## Data Availability

The data cannot be made available in the manuscript, the supplemental files, or a public repository due to data privacy agreement with the participants.
