# Supplementary material for: Patients’ perception of coercion with respect to antipsychotic treatment of psychotic disorders and its predictors
Source: Soc Psychiatry Psychiatr Epidemiol. 2021 Apr 27;56(8):1381–8. doi: 10.1007/s00127-021-02083-z (PMC8316198; doi:10.1007/s00127-021-02083-z)
Supplement: Supplementary file 1 — Supplementary file1 (DOCX 17 KB) [file 127_2021_2083_MOESM1_ESM.docx]

**Supplement “Patients’ perception of coercion with respect to antipsychotic treatment of psychotic disorders and its predictors”**

**Supplementary Material**

**Table S1.** Number of valid values

|  | Valid values | |
| --- | --- | --- |
|  | With imputed data | Without  imputed data |
|  | *N* | *N* |
| Adapted Admission Experience Scale (aAES) | 89 | 62 |
| Insight into Illness  (FKE-10) | 85 | 73 |
| Drug Attitude Inventory (DAI-10) | 88 | 66 |
| Brief Psychiatric Rating Scale, Extended Version (BPRS-24) | 77 | 65 |
| Voluntary hospital stay | 91 | 91 |
| Experience of at least one coercive measure in lifetime | 91 | 91 |
| Experience of at least one coercive measure during index hospital stay | 91 | 91 |
| Valid values (listwise) | 73 | 38 |

**Table S2.** Differences between involuntarily and voluntarily treated participants without imputations

|  | Participants | | Effect size  eta /Cohen’s *d*^(1)^ |
| --- | --- | --- | --- |
|  | Involuntarily treated  (*n* = 36)  Number (%) / mean (SD) | Voluntarily treated  (*n* = 55)  Number (%) / mean (SD) | Involuntarily treated  (*n* = 36)  Number (%) / mean (SD) |
| Experience of at least one coercive measure in lifetime | 32  (88.9%) | 27***  (49.1%) | .41 |
| Experience of at least one coercive measure during index hospital stay | 25  (69.4%) | 12***  (21.8%) | .47 |
| aAES | 12.0  (5.4) | 5.7***  (4.9) | −1.2 |
| FKE-10 | 24.0  (9.6) | 36.5***  (9.2) | 1.3 |
| DAI-10 | −3.4  (4.9) | 1.6***  (4.9) | 1.0 |
| BPRS-24 | 58.0  (17.2) | 49.8^n.s.^  (13.2) | −0.6 |

(1) Eta for frequencies; Cohen’s *d* for metric variables.

*** *p* < .001 and n.s. = not significant; Mann-Whitney U test.

**Table S3.** Correlations with perceived coercion without imputation

|  | aAES (current stay) |
| --- | --- |
| FKE-10 | - −.70** |
| DAI-10 | - −.76** |
| BPRS-24 | - .38** |

***p* < .01.

**Table S4.** Prediction of perceived coercion without imputation

| Adjusted *R*^2^ = .63 | Standardized beta |
| --- | --- |
| FKE-10 | −0.43* |
| DAI-10 | −0.44** |
| BPRS-24 | −0.04^n.s.^ |
| Coercive measure experienced during index hospital stay | 0.15^n.s.^ |
| Involuntary stay | −0.01^n.s.^ |

**p* < .05, ***p* < .01 and n.s. = not significant.
